# Bubble blasts! An adaptation for buoyancy regulation in shallow foraging gray whales

**DOI:** 10.1002/ece3.70093

**Published:** 2024-08-06

**Authors:** Clara N. Bird, Enrico Pirotta, Leslie New, K. C. Bierlich, Lisa Hildebrand, Alejandro Fernandez Ajó, Leigh G. Torres

**Affiliations:** ^1^ Geospatial Ecology of Marine Megafauna Lab, Marine Mammal Institute, Department of Fisheries, Wildlife and Conservation Sciences Oregon State University Newport Oregon USA; ^2^ Centre for Research into Ecological and Environmental Modelling University of St Andrews St Andrews UK; ^3^ Ursinus College Collegeville Pennsylvania USA

**Keywords:** baleen whales, Bayesian linear mixed effects models, behavior, diving, drones, morphology

## Abstract

Foraging efficiency is key to animal fitness. Consequently, animals evolved a variety of kinematic, morphological, physiological, and behavioral adaptations for efficient locomotion to reduce energy expenditure while moving to find, capture, and consume prey. Often suited to specific habitat and prey types, these adaptations correspond to the terrain or substrate the animal moves through. In aquatic systems, adaptations focus on overcoming drag, buoyancy, and hydrostatic forces. Buoyancy both benefits and hinders diving animals; in particular, shallow divers constantly contend with the costs of overcoming buoyancy to dive and maintain position. Pacific Coast Feeding Group (PCFG) gray whales forage in shallow habitats where they work against buoyancy to dive and feed using various foraging tactics. Bubble blasts (underwater exhalations) have been observed during several foraging tactics performed by PCFG whales. As exhalations aid buoyancy regulation in other diving animals, we hypothesize that bubble blasts are performed by longer, more buoyant whales in shallower water and that bubble blasts increase dive duration while accounting for size and tactic. We test our hypotheses using Bayesian linear mixed effects models and a 7‐year dataset of drone footage containing concurrent individual morphological and behavioral data. We find that while headstanding – a stationary, head‐down tactic – bubble blasts are performed by longer, more buoyant whales and extend the dive duration, whereas whales using forward‐swimming tactics are less likely to bubble blast. Our results suggest that PCFG gray whales may use bubble blasts as a behavioral adaption to mitigate the cost of energetically expensive tactics in their shallow habitat foraging niche.

## Graphical abstract

Gray whales use bubble blasts (underwater exhalations) to regulate their buoyancy while feeding in shallow habitats. Pacific Coast Feeding Group gray whales feed in shallow coastal habitats using a variety of behaviors. Using a 7‐year dataset of drone footage, we show that longer and more buoyant whales use bubble blasts to extend their dive duration when engaged in stationary behaviors.

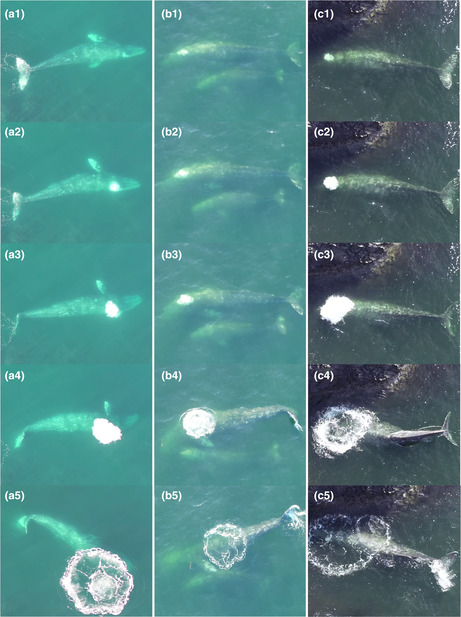

## 1 INTRODUCTION

Efficient energy acquisition and use dictates an animal's fitness (Perrigo, 1987). From internal physiological functions to behavioral needs, animals require energy to survive, forage, and reproduce. A universal energetic expense is locomotion. Consequently, animals have evolved a variety of adaptations for efficient movement within their environments. These adaptations range across kinematics (Reilly et al., 2007), morphology (Taylor, 1989; Wainwright et al., 2019), physiology (Carey, 1973; Kooyman, 1973), and behavior (Halsey, 2016; Williams, 2001).

Foraging efficiency is particularly well studied as foraging is the only behavior during which energy is both gained and spent (Norberg, 1977). In foraging theory, animals are expected to optimize the time and energy required to find and capture their prey. Profitable prey types are those with high energetic value and/or abundance that are energetically cheap to handle (Emlen, 1966). As such, many of the adaptations for efficient locomotion are associated with foraging. Body structures and gaits often correspond to specific habitats and foraging behaviors (Taylor, 1989; Woodward et al., 2007). Carnivores are classified into different categories based on their locomotor specializations and associated morphology. For example, cheetahs (*Acinonyx jubatus*) that are cursorial carnivores, have several adaptions for the high speed chases necessary to capture their prey (Wilson et al., 2018), including aerodynamic body shape and structure, heavy tails for counterbalance, and unretractable claws for traction (Eaton, 1973). In contrast, fossorial carnivores, such as American badgers (*Taxidea taxus*), have morphological adaptations better suited for digging (Taylor, 1989). Comparably, variation in the swimming gaits and morphologies of baleen whales align with their prey and foraging habitats. Blue whales (*Balaenoptera musculus*) have streamlined bodies specialized for efficient cruising with low maneuverability that allows them to efficiently travel between dispersed prey patches (Woodward et al., 2007). Gray whales (*Eschrichtius robustus*), however, are less streamlined with larger flukes and paddle‐shaped pectoral fins leading to reduced speed but increased maneuverability, which they need for their various benthic and epibenthic foraging behaviors that often involve rolling and navigating complex habitats (Woodward et al., 2007).

Marine mammals searching and foraging in highly heterogenous marine environments must engage in most behaviors while breath holding, emphasizing the need for energy efficiency. Additional energetic challenges faced by foraging marine mammals include the effects of pressure, managing oxygen stores, and the costs of moving through water. Physiologically, marine mammals can collapse their lungs to avoid decompression sickness (Kooyman, 1973) and have high levels of myoglobin in the muscle to increase oxygen availability during a dive (Kooyman & Ponganis, 1998). Morphologically, they have body structures suited for efficiently moving through water and minimizing the cost of drag (Fish & Rohr, 1999). Behaviorally, intermittent exercise is used to reduce energy expenditure (e.g., alternating between stroking and gliding (Williams, 2001)), and deep‐diving marine mammals take advantage of reduced buoyancy at depth caused by pressure and lung collapse to use the energetically cheap “glide” behavior (Williams, 1999).

Buoyancy can be both beneficial and detrimental to marine mammals. At the surface, positive buoyancy can reduce the cost of resting (Kooyman, 1973), but it is also the greatest contributor to the locomotive cost of a vertical dive from the surface to depth (Lovvorn & Jones, 1991; Stephenson et al., 1989). Furthermore, buoyancy and foraging efficiency are circularly linked; foraging success leads to increased buoyancy due to gain in lipids, which can then affect the cost of foraging and future foraging success (Adachi et al., 2014; Nousek‐McGregor et al., 2014). While deep‐diving mammals eventually escape the cost of buoyancy associated with both lung air and lipid stores past a certain depth where buoyancy forces minimize due to lung collapse and the effects of increased pressure (Williams, 2001), shallow‐diving mammals contend with this cost for the entire duration of a foraging dive (Kooyman, 1973). Sirenians have several adaptations for maintaining hydrostasis, including a horizontal diaphragm, dense bones, and skeletal weight distribution (Domning & de Buffrénil, 1991). There is also evidence that exhalation may be used to reduce the cost of buoyancy. Several pinnipeds are known to exhale prior to diving; while this behavior has been hypothesized to help prevent decompression sickness (Kooyman et al., 1972), it could also reduce buoyancy and the cost of swimming (Goldbogen et al., 2016). Furthermore, both bowhead (*Balaena mysticetus*) and sperm (*Physeter macrocephalus*) whales have been observed releasing bubbles while performing shallow resting dives, which is hypothesized to reduce buoyancy (Blackwell et al., 2022; Miller et al., 2004). However, buoyancy regulation in shallow foraging dives of marine mammals has not been well studied.

Pacific Coast Feeding Group (PCFG) gray whales forage in shallow coastal habitat typically <20 m depth (Hildebrand et al., 2021). The PCFG is a sub‐group of the Eastern North Pacific (ENP) population of gray whales (~19,260, Eguchi et al., 2024) comprising ~212 individuals (Harris et al., 2022) that forage between Northern California, USA, and Southern British Columbia, Canada during summer months (June–Nov; Calambokidis et al., 2019). Gray whales are benthic suction feeders, and in this region, PCFG whales target a variety of benthic, epibenthic, and pelagic invertebrates, including crab larvae and mysid zooplankton that aggregate near reef and rocky habitats (Darling et al., 1998; Dunham & Duffus, 2001; Hildebrand et al., 2021). Akin to their diverse diet and foraging habitat use, PCFG gray whales employ a diversity of feeding tactics identified using drone footage, including stationary headstands and side‐swims, and several forward‐moving tactics that occur mid‐water column (e.g., bottom depths of 1.9–19.7 m in the waters off central Oregon; Bird et al., 2024; Torres et al., 2018).

Moreover, there is a documented ontogenetic shift in tactic use by PCFG whales from forward‐moving behaviors into stationary behaviors that is associated with growth in length (Bird et al., 2024). Of the stationary behaviors, headstanding is the most prevalent (Bird et al., 2024). During headstanding, the whale dives down so its head is just above the bottom and maintains its body positioned vertically in the water column, often by sculling its pectoral fins or maneuvering its fluke. Maintaining a stationary position in water can be more energetically costly for diving animals than swimming forward (Iwata et al., 2017).

Headstanding may be costly because of the combined costs of foraging in such shallow habitat, particularly due to buoyancy (Lovvorn & Jones, 1991), and the cost of maintaining stationary position (Iwata et al., 2017). Therefore, given that PCFG gray whales appear to switch into this costly, yet prevalent, behavior, they may employ a behavioral adaptation to reduce the associated energetic cost, possibly by regulating their buoyancy. Interestingly, “bubble blasts” are frequently performed by foraging PCFG whales (Torres et al., 2018), which are described as “underwater release of air that rises to surface and forms a circle/puka.” The function of bubble blasts is currently unknown, but they have also been observed in gray whale wintering lagoons (Dahlheim et al., 1984) and during migration (Cummings et al., 1968), so bubble blasts may serve multiple functions depending on the season.

We hypothesize that bubble blasts associated with foraging are a behavioral adaptation used by PCFG gray whales to reduce the energetic cost of shallow foraging dives. Because buoyancy is a strong force on diving aquatic mammals, the cost of maintaining a stationary position is comparatively higher than swimming forward (Miller et al., 2004; Skrovan et al., 1999), and the use of exhalations to reduce buoyancy has been documented in other diving animals (Blackwell et al., 2022; Lovvorn & Jones, 1991; Miller et al., 2004). Furthermore, buoyancy is greater in longer individuals due to increased lung capacity (Kooyman, 1973; Sumich, 1983) and in individuals in better body condition due to the positive buoyancy of lipids (Aoki et al., 2021; Nousek‐McGregor et al., 2014). Following this theory, we hypothesize that bubble blasts will be performed by larger whales in shallower water due to increased buoyancy forces and that bubble blasts will be more likely to occur during stationary behaviors. Furthermore, we also hypothesize that a reduction in the energetic cost when a bubble blast is performed by a feeding whale will enable it to spend more time in a foraging dive (Adachi et al., 2014); therefore we expect foraging dives with bubble blasts to be longer than those without. To test these hypotheses, we use a 7‐year longitudinal dataset of drone footage of foraging PCFG gray whales to investigate: (1) the effect of bottom depth, body length and condition, and behavior on the probability of a bubble blast occurring, and (2) the effect of bubble blast occurrence on duration of a foraging dive while accounting for body length and condition, and behavior.

## 2 METHODS

### 2.1 Data collection

**FIGURE 1:**
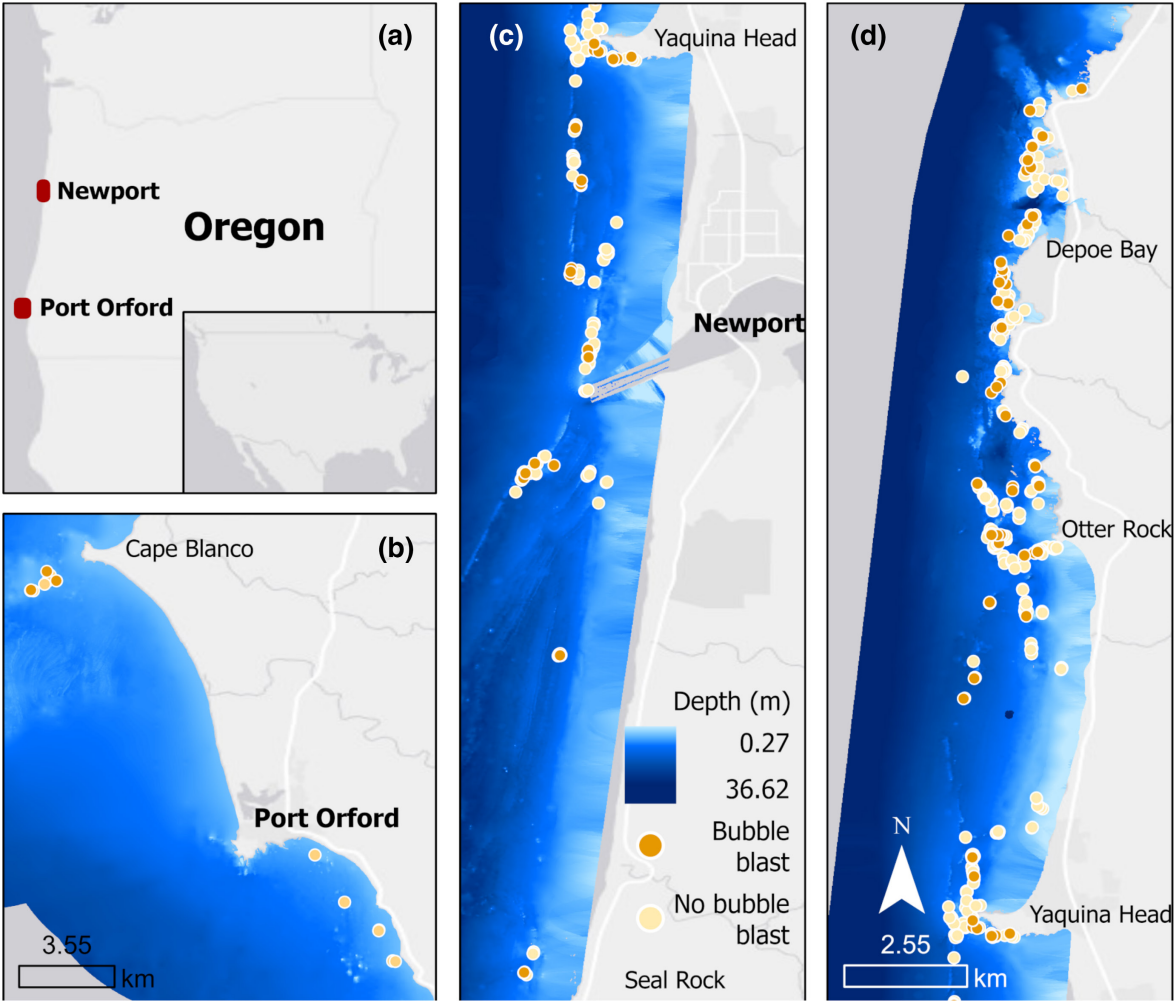
Map showing locations of foraging behavior observations in Oregon (a), specifically Port Orford (b) and Newport (c, d). Bathymetry layer and locations of foraging tactic observations colored by bubble blast occurrence are displayed for each study site (dark orange points represent tactics during which a bubble blast was performed, and light orange points represent tactics during which no bubble blast was performed).

Data was collected during boat‐based surveys off the coast of Newport (44.60765, −124.08162) and Port Orford (42.737407, −124.505301), Oregon, United States, between May and October of 2016–2022 (Figure 1). Newport and Port Orford are comparable, with both sites sharing the same prey types (Hildebrand et al., 2021, 2022) and benthic habitat substrates (Bird et al., 2024). Surveys were conducted ad libitum (Altmann, 1974) by teams of 3–4 people from a small (5.4 m) rigid hull inflatable boat in good weather (wind <22 km/h, swell <1.5 m, minimal fog or rain). When a whale was sighted, the team approached to collect photo‐identification images and conduct drone operations. During drone operations, the boat did not pursue the whale closer than 75 m to avoid any effect on behavior.

We used four different drones across the 7‐year study period: a DJI Phantom 3 Pro, 4 Advanced and 4 Pro, and an Inspire 2. The Inspire 2 was mounted with a laser altimeter (e.g., “LidarBoX”) to reduce uncertainty in photogrammetry measurements (Bierlich et al., 2024; Dawson et al., 2017). Further details on the drones used are provided in Data S1. During a flight, the pilot would locate the whale and then follow at an altitude between 20 and 40 m while the whale was visible at the surface and underwater. Video was recorded continuously for the duration of a flight (~15 min).

### 2.2 Data processing

#### 2.2.1 Behavior

Prior to annotation, drone footage was clipped to times where a whale was visible. Photo‐identification of the whale(s) in each clip was done using an aerial catalog and concurrent lateral photo‐identification images taken during the flight. Manual behavioral annotation was performed by a single experienced analyst in the open‐source Behavioral Observation Research Interactive Software (BORIS; Friard & Gamba, 2016). Each clip was reviewed at least twice per individual whale in the clip. Times when the drone was directly over the whale were also annotated in BORIS so that the GPS location of the drone during those times could be extracted to obtain GPS locations for the corresponding behavior observations.

**FIGURE 2:**
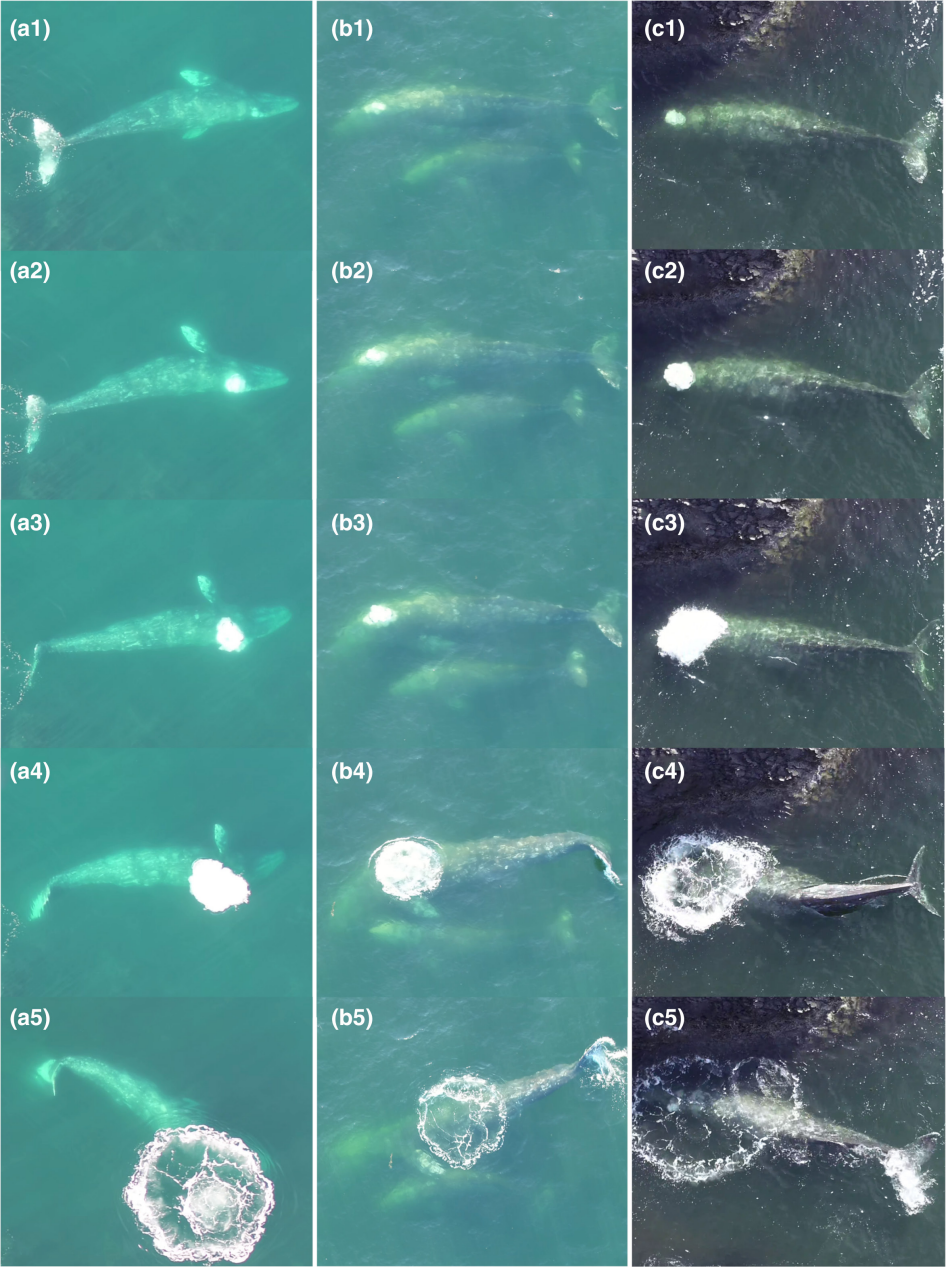
Sequential photos extracted from drone video of bubble blasts performed by PCFG gray whales during a headstand (a), side‐swim stationary (b), and subsurface feeding (c). Images 1–5 in each panel show a bubble blast event from the start of the exhalation (1) to the whale continuing to feed after the bubble has diffused at the surface (5).

Behaviors were annotated following an ethogram containing 49 behaviors (tactics: Table 1, complete ethogram in Data S1; Torres et al., 2018). For this study, we included only behaviors where bubble blasts have been observed: headstands, side‐swim (stationary), side‐swim (forward), subsurface stationary, and surface feeding. Since a bubble blast must rise to the surface, we are confident that bubble blasts were not missed, allowing us to treat events where no bubble blast was detected as absence data (Figure 2). A “tactic event” was defined here as a single instance of a foraging tactic being performed. We defined a foraging dive as a dive during which a foraging tactic occurred; the start of the dive was the start of the breath‐hold.

**TABLE 1:** Ethogram containing definitions of PCFG gray whale foraging behaviors included in this study.

Primary behavior state	Sub‐behavior tactics	Definition
Foraging	Headstand	Whale is positioned head down‐flukes up, or if in water depths less than whale body length (~12 m) whale may be more horizontal in water column; With both body positions, the whale is observed pushing head/mouth region into substrate
	Side‐swim (stationary)	Whale observed swimming on its side, but not moving forward. Characterized by frequent jaw snapping
	Side‐swim (forward)	Whale observed swimming on its side, moving forward. Characterized by frequent jaw snapping
	Upside‐down swim (forward)	Whale observed swimming upside down, moving forward. Characterized by frequent jaw snapping
	Subsurface (stationary)	Whale maintains a stationary position while feeding below the surface of the water‐oriented dorsal up. Characterized by frequent jaw snapping
	Surface feeding	Whale feeding right at the surface, frequently breaking the surface but without breathing. Characterized by frequent turning and frequent jaw snapping/flexing
	Bubble blast	Underwater release of air by whale that rises to surface and forms a circle/puka

*Note*: Complete ethogram is available in Data S1.

#### 2.2.2 Morphology

Snapshots were extracted from the drone footage to measure Total Length (TL) and Body Area Index (BAI). BAI is a standardized body condition metric that facilitates comparison between whales of different lengths, with higher values indicating better body condition (Bierlich, Hewitt, et al., 2021; Burnett et al., 2018). Whales were measured using MorphoMetriX (Torres & Bierlich, 2020) and CollatriX (Bird & Bierlich, 2020), following photogrammetry methods described in Torres et al. (2022). A Bayesian statistical model was constructed to account for photogrammetric uncertainty, returning posterior predictive distributions for each measurement (Bierlich, Schick, et al., 2021). Each individual whale was assigned one TL value per year; the latest data point within a year was selected if multiple values were available. Additional quality filtering was applied due to increased uncertainty associated with older drone models without a laser altimeter (Bierlich, Schick, et al., 2021). If an individual whale had a TL value greater than the value of a later year (suggesting the whale shrunk in size), the value was assumed to be an overestimation due to this greater photogrammetric error and instead replaced with the most temporally proximate value with the lowest standard deviation of its posterior distribution for TL. Similarly, if a TL value was more than a meter larger than a value from the previous year, and the individual had not been a calf, it was assumed to be an overestimation and replaced with the most temporally proximate value with the lowest standard deviation of its posterior distribution for TL. If available, the BAI measurement from the date of the behavior observation was selected; if not, the nearest measurement within a 2‐week window (on either side) was applied (Pirotta et al., 2023).

#### 2.2.3 Bathymetry

For the Newport study site, we used a high‐resolution (<10 m^2^) bathymetry map developed for the area (Bird et al., 2024). Bathymetry for the Port Orford study area was extracted from a NOAA coastal bathymetry layer. Newport and Port Orford have similar bathymetries; the mean bottom depths for Newport and Port Orford were 8.6 m (range: 3.03–21.5 m) and 10 m (range: 3.2–20.7 m), respectively. Bottom depth values were associated with tactic events using the drone's GPS location at that time. The bottom depth values at those points were extracted and averaged across the duration of the tactic event. Finally, the tide level at that hour was added to the bottom depth values to calculate the bottom depth at the time the tactic was performed.

### 2.3 Statistical analysis

Analyses were performed in R v4.1.2 (R Core Team, 2021) using *RStan* (Stan Development Team, 2020), and *rethinking* (McElreath, 2020). All data and code are available at: https://figshare.com/s/09629fb692a56322f390.

#### 2.3.1 Probability of bubble blasts occurring

We used Bayesian mixed‐effects logistic regression models to explore the relationship between bubble blast occurrence and individual morphology, bathymetry, and tactic. The binary response was whether a bubble blast occurred during a tactic event. Buoyant force is affected by the pressure from the water above the whale (greater pressure results in less buoyant force); therefore, only the depth at which a whale is feeding is related to buoyancy. Feeding depth is not available from drone observations, except for headstanding, which is the only tactic where the whale's body is vertical in the water column with its head at the bottom, i.e., where bottom depth represents feeding depth. Consequently, we split the dataset by tactic and ran two separate models, with and without bottom depth as a term. The first model, the “headstanding model,” included TL, BAI, and bottom depth as fixed effects. The second model, the “non‐headstanding model,” included TL, BAI, and tactic (side‐swim (stationary), side‐swim (forward), subsurface stationary, or surface feeding) as fixed effects. Both models included an individual‐level random effect to account for multiple observations of an individual, and a sighting‐level random effect to capture the conditions at the sighting that could affect observation bias such as glare, watercolor, and weather conditions. Photogrammetric uncertainty was incorporated in the Bayesian analysis by imputing the estimated true BAI and TL from the posterior distribution of each measurement. All continuous variables were z‐score standardized. The prior distributions used for all model parameters are reported in Table 3.

For both models, we ran three chains of 300,000 iterations, with the first 100,000 used as warm‐up. We assessed model convergence using effective sample size, $\hat{R}$ values, and visual examination of trace plots (McElreath, 2020). We assessed model fit by first calculating the ROC and selecting a threshold using the Youden method using the *pROC* package (Robin et al., 2011). We then applied the threshold to classify the predicted data and create a confusion matrix using the caret package (Kuhn, 2008).

#### 2.3.2 Tactic duration

We used a Bayesian linear mixed‐effects model to assess the effect of TL, BAI, tactic, and bubble blast occurrence on tactic duration. The response was the natural log of the tactic event duration in seconds. The natural log‐transformed data were used because generalized linear models (GLMs) using the untransformed data failed to meet the assumptions of simple linear regression. We included TL, BAI, tactic, and bubble blast occurrence as fixed effects, with interactions between each tactic and bubble blast occurrence. We also included an individual‐level random effect to account for repeat samples and any unquantified differences in individual physiology that could affect tactic duration. Photogrammetric uncertainty was incorporated in the Bayesian analysis by imputing the estimated true BAI and TL from the posterior distribution of each measurement. All continuous variables were z‐score standardized. The prior distributions used for all model parameters are reported in Table 3.

We ran three chains of 30,000 iterations with the first 10,000 as warm‐up. Model convergence was assessed using effective sample size, $\hat{R}$ values, and visual examination of trace plots (McElreath, 2020). Model fit was assessed using posterior predictive checks, bayes *R* ^2^ (Gelman et al., 2019), and visual inspection of the residuals.

## 3 RESULTS

We observed 645 foraging tactic events performed by 75 individual whales; of these 75 individuals, 39 were observed in 1 year, 21 were observed in 2 years, 11 were observed in 3 years, three were observed in 4 years, and one was observed in 5 years. These observations were made across 196 sightings; of these, 144 were single whale sightings, 35 were of two whales, seven were of three whales, three were of four whales, and one was of five whales. Of the 35 two whale sightings, six were mom‐calf pairs. Bubble blasts were observed during 87 of the 645 foraging tactic events. A bubble blast was typically performed, on average, 27 s (CI_95_: 8.60, 86.00) after the start of a foraging dive. Most bubble blasts were observed during headstands (*n* = 58), but headstanding is also the most frequently observed tactic (*n* = 331). Proportionally within each tactic, bubble blasts were most common during side‐swim (stationary), occurring in 21.7% of events, followed by headstanding (17.5%), surface feeding (16.7%), subsurface stationary (12.8%), and side‐swim (forward) (2.8%) (Table 2).

**TABLE 2:** Number of PCFG gray whale foraging tactic events where a bubble blast was observed or not observed, by tactic.

	Bubble blast	No bubble blast
Headstand	58 (0.175)	273 (0.825)
Side‐swim (forward)	5 (0.028)	174 (0.972)
Side‐swim (stationary)	13 (0.217)	47 (0.783)
Subsurface stationary	5 (0.128)	34 (0.872)
Surface feeding	6 (0.167)	30 (0.833)

*Note*: The values in parentheses represent the proportion of times a bubble blast was or was not observed during each tactic.

### 3.1 Probability of bubble blasts occurring

#### 3.1.1 Headstanding model

**FIGURE 3:**
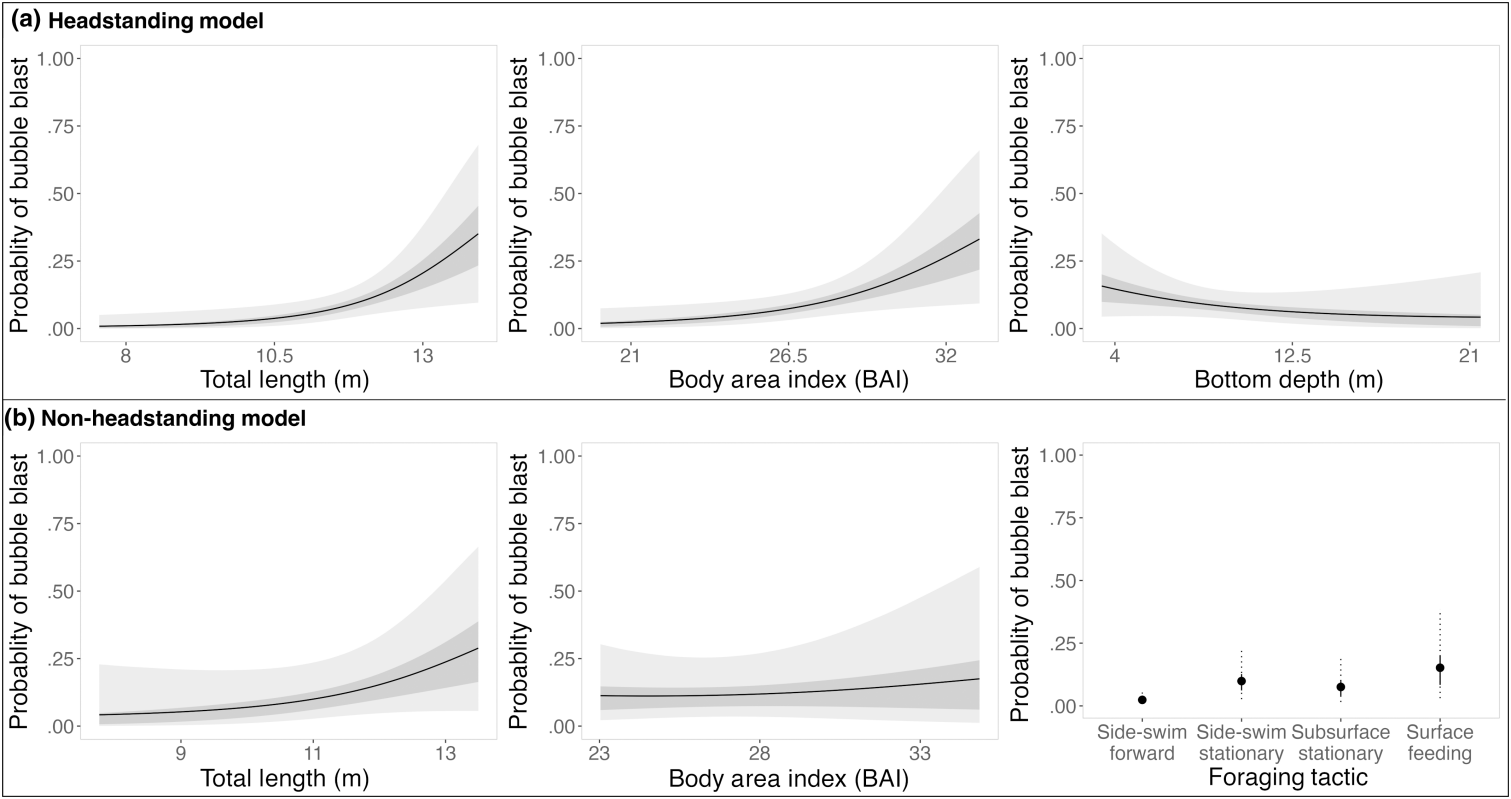
Model predictions for the probability of a bubble blast occurrence during PCFG gray whale foraging tactic for (a) the “headstanding model” testing for the effect of total length (TL), Body Area Index (BAI), and depth on the probability of a bubble blast being observed during headstanding; and (b) the “non‐headstanding model” testing for the effect of TL, BAI, and tactic on the probability of a bubble blast being observed during a non‐headstanding event. In the first five panels, the line represents the posterior mean probability, the darker‐shaded region represents the 50% credible interval, and the lighter‐shaded region represents the 95% credible interval. In the last panel, the points represent the posterior mean probabilities, the solid lines represent the 50% credible intervals, and the dashed lines represent the 95% credible intervals.

**FIGURE 4:**
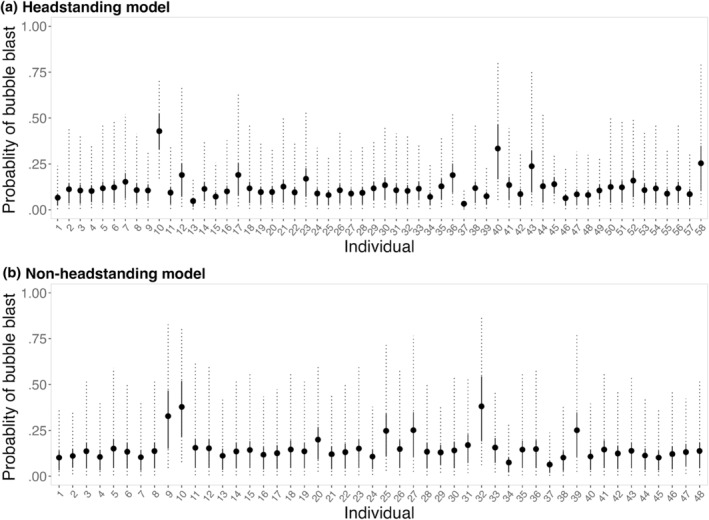
Model predictions for the individual random effect in the (a) headstanding and (b) non‐headstanding models of the probability of bubble blast occurrence during PCFG gray whale foraging. The points represent the posterior mean probabilities, the solid lines represent the 50% credible intervals, and the dashed lines represent the 95% credible intervals.

The probability of a bubble blast occurring during headstanding increased with both TL and BAI, while bottom depth only had a weak negative effect (Figure 3a, Table 3). The 95% credible intervals for TL and BAI did not overlap with 0. Specifically, the probability of a bubble blast occurring during headstanding increased with both TL (odds ratio: 2.341, CI_95_: 1.168, 4.333) and BAI (odds ratio: 1.969, CI_95_: 1.105, 3.384). Moreover, bubble blast occurrence decreased with bottom depth (odds ratio: 0.793, CI_95_: 0.485, 1.194). The estimated probability of decrease was 87%, although the credible interval overlaps with 1. There was also variation in the individual random effect, where some individuals were more likely to perform bubble blasts than others (Figure 4a). For the ROC, the area under the curve was 0.93 and the selected threshold was 0.17. The resulting confusion matrix yielded an accuracy of 81%. Prior sensitivity analysis indicated that the variance of the posterior distributions increased slightly with a more relaxed prior. However, the distributions all shifted in the direction of the effect, and the posterior estimates of the means remained relatively unchanged. The results presented all use the priors listed in Table 3, as these proved to be slightly more conservative when determining the potential effect of the explanatory variables.

**TABLE 3:** Model coefficients for all three models.

Name	Coefficient	Prior	Headstanding model	Non‐headstanding model	Tactic duration model
			Mean (SD)	Mean (SD)	Mean (SD)
Intercept	a	Normal (0,1)	−2.400 (0.361)	−2.157 (0.574)	3.419 (0.080)
Depth	bD	Normal (0,1)	−0.257 (0.229)	–	–
BAI	bB	Normal (0,1)	0.636 (0.285)	0.066 (0.318)	−0.067 (0.052)
TL	bT	Normal (0,1)	0.795 (0.333)	0.609 (0.427)	0.055 (0.064)
Julian Day	bJD	Normal (0,1)	0.147 (0.277)	−0.010 (0.315)	0.038 (0.049)
Side‐swim forward	bSSF	Normal (0,1)	–	−1.683 (0.602)	−0.634 (0.102)
Side‐swim stationary	bSSS	Normal (0,1)	–	−0.189 (0.617)	−0.541 (0.150)
Subsurface stationary	bSBS	Normal (0,1)	–	−0.545 (0.668)	−0.540 (0.178)
Surface feeding	bSF	Normal (0,1)	–	0.253 (0.744)	0.109 (0.222)
Individual level random effect	sigma_id	Uniform (0,10)	1.218 (0.393)	1.215 (0.524)	0.350 (0.065)
Sighting random effect	sigma_dtst	Uniform (0,5)	1.086 (0.465)	0.819 (0.489)	–
Interaction term between bubble blast and side‐swim forward	bBB_SSF	Normal (0,1)	–	–	−0.090 (0.400)
Interaction term between bubble blast and side‐swim stationary	bBB_SSS	Normal (0,1)	–	–	0.199 (0.301)
Interaction term between bubble blast and subsurface stationary	bBB_SBS	Normal (0,1)	–	–	0.049 (0.429)
Interaction term between bubble blast and surface feeding	bBB_SF	Normal (0,1)	–	–	−0.572 (0.410)

*Note*: A dash indicates that the variable was not included in the model.

#### 3.1.2 Non‐headstanding model

There was no clear evidence for an effect of TL, BAI, and most of the non‐headstanding tactics on bubble blast occurrence (Figure 3b, Table 3). The only exception was with side‐swimming forward, which had a decreased probability of a bubble blast occurring (odds ratio: 0.222, CI_95_: 0.056, 0.599). Moreover, bubble blast occurrence increased with TL (odds ratio: 2.019, CI_95_: 0.821, 4.422). The estimated probability of increase was 93%, although the credible interval overlaps with 0. The 95% credible interval for the effect of BAI overlapped with 0 (coefficient: 0.066, CI_95_: −0.568, 0.700). Some individuals were also more likely to bubble blast, as shown by the individual random effect (Figure 4b). For the ROC, the area under the curve was 0.93 and the selected threshold was 0.17. The resulting confusion matrix yielded an accuracy of 82%. Prior sensitivity analysis indicated that there was not enough information in the data to inform any relationship regarding bSSF, bSSS, bSBS, and bSF. However, while the variance of the posterior distributions for the other parameters increased slightly with a more relaxed prior, the distributions all shifted in the direction of the effect, and the posterior estimates of the means remained relatively unchanged. The results presented all use the priors listed in Table 3, as these proved to be slightly more conservative when determining the potential effect of the explanatory variables.

### 3.2 Tactic duration model

**FIGURE 5:**
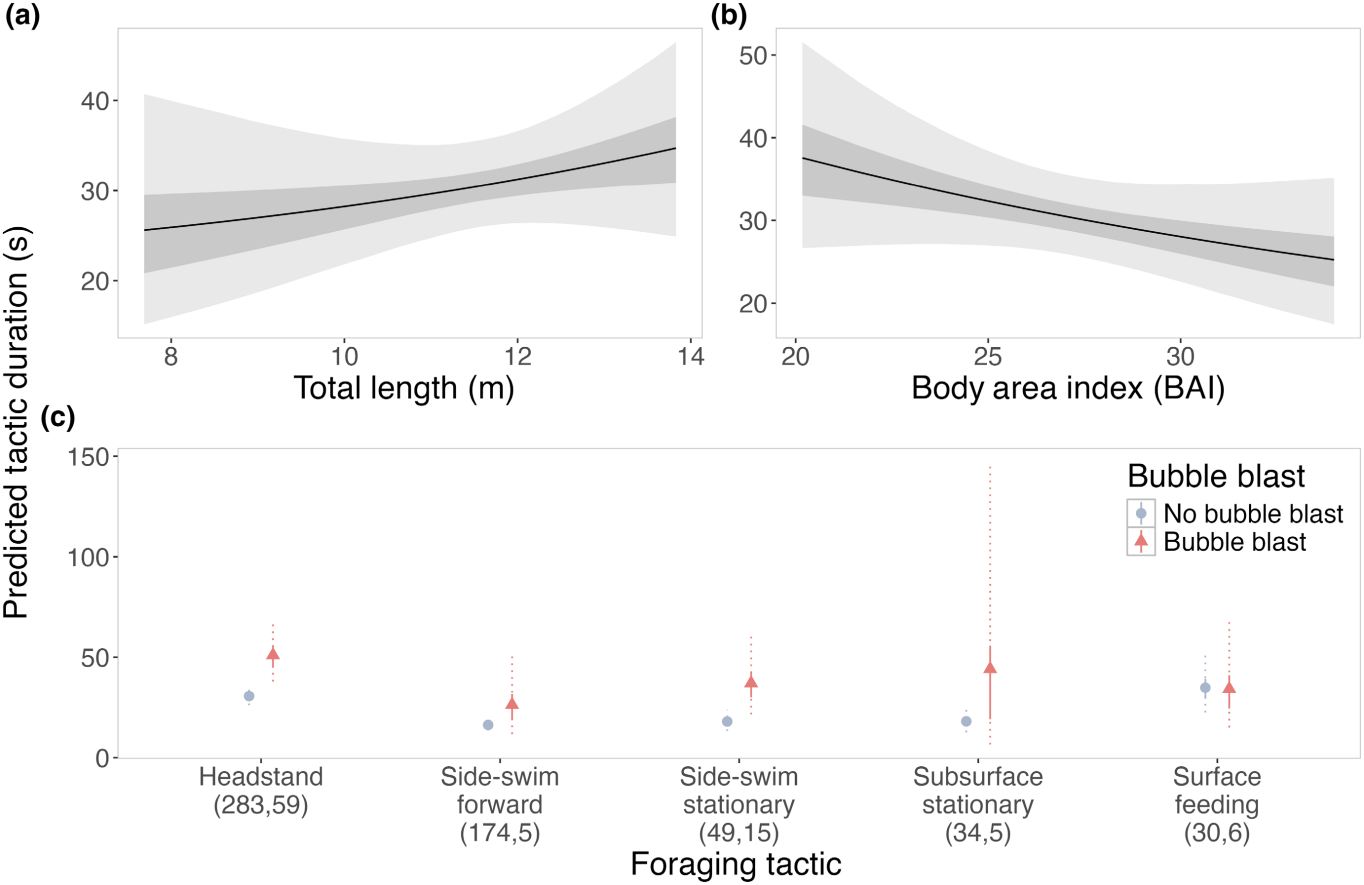
Model predictions for the effects of PCFG gray whale (a) total length (TL), (b) Body Area Index (BAI), and (c) tactic and bubble blast occurrence on the duration of a tactic event, transformed back into seconds from the log scale. In panels (a) and (b), the line represents the posterior mean probability, the darker‐shaded region represents the 50% credible interval, and the lighter‐shaded region represents the 95% credible interval. In panel (c), the points represent the posterior mean probabilities, the solid lines represent the 50% credible intervals, and the dashed lines represent the 95% credible intervals. Gray circles indicate that no bubble blast occurred, while orange triangles indicate the occurrence of a bubble blast. The sample sizes per tactic are reported in parentheses under the tactic name: The first value indicates the number of observations with no bubble blast for that tactic, and the second value indicates the number of observations with a bubble blast for that tactic.

Bubble blast occurrence had a positive effect on the duration of headstanding and side‐swimming stationary. The 95% credible interval of the effect of both TL and BAI on the natural log of the duration of a foraging tactic overlapped with 0 (Table 3), so there was a less identifiable effect. The log duration increased with TL (0.055, CI_95_: −0.073, 0.180) and the estimated probability of increase was 80% (Figure 5a). Conversely, the log duration decreased with BAI (−0.067, CI_95_: −0.169, 0.033) and the estimated probability of decrease was 90% (Figure 5b). The occurrence of a bubble blast was associated with the log duration of headstands and the side‐swimming stationary tactic (Figure 5c): when transformed back to the scale of seconds, a bubble blast increased the predicted duration of headstanding and side‐swimming stationary by 20.4 s (CI_95_: 7.967, 36.053) and 19.1 s (CI_95_: 3.334, 42.197), respectively. Assessment of the residual plots indicated that model assumptions had been met, and the Bayesian *p*‐values were .47 and .62 for the replicated data and standard deviations, respectively (a value close to 0.5 indicates a good fit (Bayarri & Berger, 2000)). The Bayes *R* ^2^ was .275 (CI_95_: 0.219, 0.328). Prior sensitivity analysis showed no real prior sensitivity to more relaxed priors.

## 4 DISCUSSION

Our study assessed the hypothesis that bubble blasts are a behavior used by PCFG gray whales to regulate buoyancy and thus make foraging more energetically efficient. We tested how total length, body condition, and behavior affected the probability of bubble blast occurrence and how total length, body condition, behavior, and bubble blast occurrence affected foraging dive duration. We found support for our hypothesis as longer and more buoyant whales were more likely to bubble blast while headstanding, and the duration of headstanding and side‐swim stationary dives increased when a bubble blast was performed. We also found individual variation in the probability of bubble blast occurrence, suggesting that this behavior could be a specialization.

The positive relationships between TL and BAI and the probability of bubble blast occurrence align with our hypothesis that bubble blasts are performed by larger whales due to increased buoyancy forces. Specifically, longer whales have larger lungs that hold more air (Sumich, 1983) and are more buoyant (Kooyman, 1973), and baleen whale body condition is linked to blubber thickness that contains positively buoyant lipids (Aoki et al., 2021; Nousek‐McGregor et al., 2014). The weak negative relationship between bottom depth and the probability of a bubble blast during headstanding (with a 95% credible interval that overlapped with zero) may reflect that even the deepest bottom depth in this habitat (~20 m) is still shallow for a gray whale, meaning that the effect of buoyancy may not change significantly within the depth range in this study area. The lack of relationship between TL and BAI and the probability of bubble blasting during non‐headstanding tactics could be due to sample size; in absolute numbers, fewer bubble blasts occurred during the non‐headstanding tactics and the non‐headstanding tactics themselves are less common. The negative relationship between side‐swimming forward and the probability of a bubble blast is logical given that buoyancy has less of an effect on whales moving compared to holding stationary (Skrovan et al., 1999).

The positive relationship between bubble blast occurrence and the natural log of the tactic dive duration indicates that bubble blasts allow individuals to extend dive time. This extension suggests that bubble blasts may facilitate oxygen conservation by reducing the cost of locomotion during the dive and improving energetic efficiency. Furthermore, we found a 90% probability of a negative relationship between BAI and the natural log of the tactic dive duration, indicating that whales in higher body condition (i.e., more buoyant) have reduced tactic dive durations. As whales in higher body condition are also more likely to bubble blast, these combined results could suggest that gray whales in high body condition use bubble blasts to extend dive times that are reduced by increased buoyant forces associated with body condition. Similarly, increased locomotive efficiency due to lipid store‐related buoyancy changes in northern elephant seals (*Mirounga angustirostris*) is linked to improved dive efficiency, quantified as longer time spent foraging at depth (Adachi et al., 2014). Marine mammals display other behavioral adaptations to improve energetic efficiency by reducing cost of transport. Several cetacean species use intermittent exercise as an efficient locomotive behavior to prolong dive duration (Williams, 1999). Rorquals with low maneuverability use behavioral changes to improve turning efficiency (Segre et al., 2022). Additionally, pinnipeds alter dive characteristics including surface intervals (Cornick et al., 2006) or dive angle (Boyd et al., 1997) to improve swimming efficiency. It's been estimated that oxygen extraction in gray whales occurs in the first 20 to 30 s following an inhalation (Sumich, 2001), therefore the delay of about 27 s between the last breath and the bubble blast suggests the possibility that gray whales exhale after allowing time for some gas exchange to occur, increasing oxygen stores.

Our results on the patterns of bubble blast use by foraging PCFG gray whales align with the proposed use of bubble releases for buoyancy regulation in several diving aquatic animals, including resting sperm and bowhead whales (Blackwell et al., 2022; Miller et al., 2004) and ducks (*Aythya* spp.; Butler & Woakes, 1979). Therefore, bubble releases may be a shared strategy across several air‐breathing aquatic animals to manage the cost of buoyancy while diving.

Our finding of increased bubble blast probability with increased body condition may explain why Bird et al. (2024) found that body condition had no effect on the probability of any foraging tactic being used. If gray whales can use bubble blasts to overcome buoyancy, foraging tactic choice does not need to be linked with current body condition. Furthermore, the use of bubble blasts means that, up to a certain point, buoyancy regulation allows whales to continue feeding in this habitat even while gaining lipid reserves and consequently buoyancy. PCFG gray whales feed in water considerably shallower than that of the rest of the ENP (~20 vs. ~90 m), meaning that they likely encounter higher buoyancy costs than foraging ENP whales. Bubble blasts may be a behavioral adaptation that enables these PCFG whales to cost‐effectively feed in such shallow habitat.

While this study supports our hypothesis that bubble blasts are used for buoyancy regulation, it is possible that there are alternate or additional functions for this behavior. One alternate theory is that bubble blasts are used for prey manipulation, akin to how humpback whales (*Megaptera novaeangliae*) use bubbling to corral prey (Hain et al., 1982). While this theory is possible, gray whales are not engulfment foragers, and they do not appear to chase their prey after producing a bubble blast nor do they aim the bubble towards the location where they ultimately feed (Figure 2). Therefore, it seems unlikely that bubble blasts serve a corral function.

A second alternative purpose could be communication. It has been theorized that dwarf minke whales (*Balaenoptera acutorostrata*) may use bubble blasts for antagonistic communication (Birtles et al., 2002) and hippopotami (*Hippopotamus amphibius*) may use bubble blasts as an amphibious call in response to disturbance (Barklow, 2004). Considering that bubble blasts have been performed by gray whales in wintering lagoons (Dahlheim et al., 1984) and while migrating (Cummings et al., 1968), it seems plausible that communication could be a function of bubble blasts as well. Further work recording the reactions of PCFG gray whale prey, and other gray whales to a bubble blast is needed to fully understand this behavior.

Subsequent studies should also quantify PCFG gray whale foraging efficiency and the possible role of behavioral innovation and social transmission. While we found that dive duration increased, we could not quantify how much prey was consumed and whether longer dives corresponded to increased prey consumption. Moreover, other factors could affect dive duration including variable pre‐dive inhalation volume, prey abundance and distribution, disturbance, and other individual physiological traits. The presence of individual variation in the probability of bubble blast occurrence could indicate that bubble blasts are a behavioral innovation that is spreading through the study group through some form of social learning (Wild et al., 2020).

In conclusion, we present evidence that longer PCFG gray whales in higher body condition are more likely to use bubble blasts and that stationary foraging dives during which a bubble blast is performed are longer in duration; these results suggest that bubble blasts may reduce the energetic costs of their stationary, shallow water foraging dives. This result can help us better quantify the costs of foraging, which are important to understand how changes in behavior patterns, either caused by environmental change or disturbance, may affect energy expenditure. We also contribute to the limited body of work studying the behavior and energetic costs of shallow water foraging by air‐breathing animals, demonstrating the utility of drones for collecting concurrent and replicate morphology and behavior data of individuals. While the adaptations of deep‐diving marine mammals have been well documented, shallow water diving presents its own challenges that marine mammals may adapt to behaviorally to increase access to diverse foraging niches.

## AUTHOR CONTRIBUTIONS

**Clara N. Bird:** Conceptualization (equal); data curation (equal); formal analysis (lead); investigation (equal); methodology (lead); visualization (lead); writing – original draft (lead); writing – review and editing (lead). **Enrico Pirotta:** Formal analysis (supporting); methodology (supporting); writing – review and editing (equal). **Leslie New:** Formal analysis (supporting); funding acquisition (supporting); methodology (supporting); writing – review and editing (equal). **K.C. Bierlich:** Data curation (equal); investigation (equal); writing – review and editing (equal). **Lisa Hildebrand:** Investigation (equal); writing – review and editing (equal). **Alejandro Fernandez Ajó:** Investigation (equal); writing – review and editing (equal). **Leigh G. Torres:** Conceptualization (equal); funding acquisition (lead); investigation (lead); supervision (equal).

## CONFLICT OF INTEREST STATEMENT

The authors declare no competing interests.

### OPEN RESEARCH BADGES

This article has earned Open Data and Open Materials badges. Data and materials are available at https://figshare.com/s/09629fb692a56322f390.

## Supporting information


Data S1:

## Data Availability

Data and code available from FigShare https://figshare.com/s/09629fb692a56322f390.
