# Supplementary material for: Bubble blasts! An adaptation for buoyancy regulation in shallow foraging gray whales
Source: Ecol Evol. 2024 Aug 6;14(8):e70093. doi: 10.1002/ece3.70093 (PMC11301446; doi:10.1002/ece3.70093)
Supplement: Supplementary file 1 — Data S1: [file ECE3-14-e70093-s001.docx]

**Supplementary Materials for Bubble blasts! An adaptation for buoyancy regulation in shallow foraging gray whale**

Table of Contents

[Ethogram 2](#_Toc157088344)

[Drone Details 4](#_Toc157088345)

[References 5](#_Toc157088346)

# Ethogram

*Table S1. Complete ethogram of all behaviours used to annotate footage. Taken from supplementary materials for Bird et al. (in review).*

| **Primary behaviour state** | **Sub-behaviour tactics** | **Point or State** | **Definition** |
| --- | --- | --- | --- |
| Foraging |  |  |  |
|  | Headstand | State | Whale is positioned head down-flukes up, or if in water depths less than whale body length, whale may be more horizontal in water column; With both body positions the whale is observed pushing head/mouth region into substrate. |
|  | Side-swim (stationary) | State | Whale observed swimming on its side, but not moving forward. Characterised by frequent jaw snapping. |
|  | Side-swim (forward) | State | Whale observed swimming on its side, moving forward. Characterised by frequent jaw snapping. |
|  | Upside-down swim (forward) | State | Whale observed swimming upside-down, moving forward. Characterised by frequent jaw snapping. |
|  | Subsurface (forward) | State | Whale swims subsurface while feeding. Characterised by frequent jaw snapping. |
|  | Subsurface (stationary) | State | Whale maintains a stationary position while feeding below the surface of the water oriented dorsal up. Characterised by frequent jaw snapping. |
|  | Surface feeding | State | Whale feeding right at the surface, frequently breaking the surface but without breathing. Characterized by frequent turning and frequent jaw snapping/flexing. |
|  | Skim feeding | State | Whale swims at the surface with mouth open for an extended period. Characterised by moving forward in a straight line. |
|  | Bubble blast | Point | Underwater release of air by whale that rises to surface and forms a circle/puka. |
|  | Bubbles from mouth | Point | A stream of bubbles is observed emanating from the whale's mouth while it is at the surface. |
|  | Open and closes mouth under water | Point | Mouth opens and closes in quick succession, like taking a bite. |
|  | Open mouth at surface | State | The mouth of the whale is observed at the surface (so that the baleen is visible). |
|  | Sediment from mouth | Point | A stream of sediment is observed emanating from the whale's mouth. |
|  | Sediment from unknown source | Point | Sediment observed coming from unknown source, whale is headstanding but mouth not visible. |
|  | Sharking | State | Whale's fluke observed above the water surface. |
| Social |  |  | Whales interacting with each other, usually involves some form of tactile interaction. |
|  | Bump-tactile interaction | Point | Two adult whales are observed making body contact. |
|  | Pair coordinated surfacings | State | Two whales surface together in close proximity multiple times (not a mother-calf pair). |
|  | Promiscuous behaviour | State | A whale positions itself upside-down underneath the ventral side of another whale. |
|  | Echelon swimming | State | Mother and calf observed swimming very closely together. |
|  | Mother-calf tactile interaction | State | Mother and calf whales are observed touching each other through any body part (head, pectoral fins, body). |
|  | Presumed Nursing | State | Calf is observed going into a nursing position under its mother's ventral surface and positioning there for an extended period. |
|  | Pass under | State | Calf observed crossing under mother briefly so clearly not nursing. |
|  | Pass over | State | Calf observed crossing over mother. |
| Rest |  |  | Logging type behaviour observed where whale remains in same location, lying at or just below the surface, and with minimal to no active fluking to promote movement. Surfacings are generally slow and at regular intervals. |
| Travel |  |  | Whale shows directed travel in a consistent direction, with regular surfacing intervals. |
| Other |  |  |  |
|  | Body Roll | Point | Whale observed moving its body in a barrel roll. |
|  | Defecate | Point | Faeces is observed streaming from posterior end of whale (care is taken to avoid confusion with sediment emanating from mouth. |
|  | Lower jaw flex | Point | Lower jaw of whale is quickly pushed/flared outward. |
|  | Pec flare | Point | The whale swings its pectoral fin outward. |
|  | Turning | State | Whale makes rapid change in direction (over a spatial scale of <20 m). |
|  | Blow | Point | Whale exhales and inhales. |
|  | Breach | Point | Whale is observed breaching out of the water. |
|  | Dive | Point | Whale dives to begin a long breath hold duration. |
|  | Fluke swish | Point | Rapid "swish" movement of fluke horizontally from side to side. |
|  | Swimming at surface | State | Whale is swimming slowly at the surface performed short breath hold dives in-between blows. |
|  | Sculling | State | Whale observed rotating pectoral fins in circles. |
|  | Spyhopping | State | Whale observed lifting head vertically out of the water. |
|  | Swerve | Point | Whale observed tilting/partially rolling to the side but not completely. |

A video compilation of the foraging tactics is available here: <https://figshare.com/s/09629fb692a56322f390>

# Drone Details

We used several drone models for data collection between 2016-2022 (Table S2). Each drone was piloted using manual remote flight control and real-time camera output through a tablet ground station. Each camera were stabilized by a 3-axis brushless gimbal. Video output was recorded at 4K and a 1080 p down sample was transmitted real-time to the pilot at 30 Hz. While each drone was equipped with a barometer for measuring altitude, the DJI Inspire 2 also contained a LiDAR altimeter (e.g., “LidarBoX” Bierlich et al., 2024) (Table S2). Taken from supplementary materials for Bird et al. (*in review*).

*Table S2. Camera specifications associated with each unoccupied aircraft system (UAS) used in this study. Each UAS had a barometer to record the altitude of the drone during video collection, while the Inspire 2 also had an altimeter (LiDAR) (Dawson et al. 2017; Bierlich et al. 2024)*

| **UAS** | **Years used** | **Sensor (mm)** | **Pixel resolution (pox)** | **Focal length lens (mm)** | **Altimeter** |
| --- | --- | --- | --- | --- | --- |
| Phantom 3 Pro | 2016, 2017 | 6.16 x 4.6 | 3840 x 2160 | 3.61 | Barometer |
| Phantom 4 | 2016, 2017 | 6.16 x 4.6 | 3840 x 2160 | 3.61 | Barometer |
| Phantom 4 Pro | 2017, 2018, 2019 | 13.2 x 8.8 | 3840 x 2160 | 8.8 | Barometer |
| Inspire 2 | 2020, 2021, 2022 | 17.3 x 13 | 3840 x 2160 | 25 | Barometer & LiDAR |

# References

Bierlich KC, Wengrove D, Bird CN, Davidson R, Chandler T, Torres LG, Cantor M. 2024. LidarBoX: a 3D-printed, open-source altimeter system to improve photogrammetric accuracy for off-the-shelf drones. Drone Syst Appl. 12:1–10. doi:10.1139/dsa-2023-0051.

Dawson SM, Bowman MH, Leunissen E, Sirguey P. 2017. Inexpensive Aerial Photogrammetry for Studies of Whales and Large Marine Animals. Front Mar Sci. 4(NOV). doi:10.3389/fmars.2017.00366. https://www.frontiersin.org/article/10.3389/fmars.2017.00366/full.
